# Hematoma, Perihematomal Edema, and Total Lesion Predict Outcome in Patients With Intracerebral Hemorrhage

**DOI:** 10.1002/brb3.70340

**Published:** 2025-02-18

**Authors:** Qin Huang, Lin Wu, Ziwei Song, Zhi Zhang, Hongla Kuang, Yuping Zhu, Chenying Zeng, Lanjiao Zhang, Hudie Zhang, Zubing Xu, Wenyuan Xu, Jing Lin

**Affiliations:** ^1^ Department of Neurology, The First Affiliated Hospital, Jiangxi Medical College Nanchang University Nanchang China; ^2^ Institute of Neurology, Jiangxi Academy of Clinical Medical Science, The First Affiliated Hospital, Jiangxi Medical College Nanchang University Nanchang China; ^3^ Rare Disease Center, The First Affiliated Hospital, Jiangxi Medical College Nanchang University Nanchang China; ^4^ Key Laboratory of Rare Neurological Diseases of Jiangxi Provincial Health Commission, Jiangxi Medical College Nanchang University Nanchang China; ^5^ Department of Radiology, The First Affiliated Hospital, Jiangxi Medical College Nanchang University Nanchang China

**Keywords:** intracerebral hemorrhage, perihematomal edema, predict, relative perihematomal edema, total lesion

## Abstract

**Background:**

The present study aimed to evaluate the predictive abilities of hematoma volume, perihematomal edema (PHE) volume, and total lesion (hematoma + PHE) volume for poor outcome in patients with intracerebral hemorrhage (ICH).

**Methods:**

Patients admitted to our department between January 2015 and March 2023 were retrospectively enrolled according to the inclusion criteria and exclusion criteria. Demographic characteristics, clinical information, laboratory examinations, and imaging data were collected.

**Results:**

We included 510 patients with initial computerized tomography (CT) scan (342 [67.1%] male, median age = 62 years); 142 patients had CT scans at admission and 72 h post ICH, and 350 patients had CT scans at admission and 5–9 days after onset. Multivariate logistic regression analysis revealed that absolute hematoma, absolute PHE, and absolute total lesion at admission; absolute hematoma and absolute total lesion at 72 h after onset; absolute hematoma, absolute PHE, and absolute total lesion at 5–9 days post ICH were independently related to poor outcome (*p* < 0.05). Furthermore, receiver operating characteristic curves demonstrated that the total volume of hematoma and PHE at 5–9 days post ICH was a better indicator to predict poor outcome, compared to other risk factors in patients with ICH (area under curve = 0.778, 95%CI: 0.729–0.826).

**Conclusion:**

The total volume of hematoma and PHE at 5–9 days after onset had the highest ability in predicting poor outcome in patients with ICH.

## Graphical abstract

This study demonstrated the total volume of hematoma and perihematomal edema at 5–9 days after onset had the highest ability in predicting poor outcome in patients with intracerebral hemorrhage.

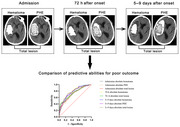

## 1 Introduction

Stroke is the main cause of death and disability in the world (Tu et al. 2023; Tsao et al. 2022). Intracerebral hemorrhage (ICH), as the second common subtype of stroke, is responsible for 10%–15% of the 15 million strokes worldwide each year (Venkatasubramanian et al. 2013). Patients with ICH often experience a poor functional outcome, with only 12%–39% of patients living independently at 6 months after onset (van Asch et al. 2010). Despite these devastating outcomes, the current medical or surgical therapeutic options for ICH are very limited. Therefore, it is necessary to identify reliable prognostic factors for ICH and provide new therapeutic targets.

Outcome after ICH is a complex subject and may be affected by multiple factors, through diverse perspectives, and at different time points. It is well known that some traditional factors affecting the prognosis of ICH include the severity of symptoms at onset, age, and hyperglycemia (Rost et al. 2008; Lusk et al. 2023). Additionally, previous studies have suggested that hematoma volume and location are strongly associated with functional outcome and mortality in patients with ICH (Pinho et al. 2019). More recently, perihematomal edema (PHE), as the radiological manifestation of secondary injury, has been reported an association with worsened outcome in ICH patients (Murthy et al. 2015; Wu et al. 2017; Volbers et al. 2016). However, the volume of hematoma, PHE, and total volume at different time points after ICH have varying effects on the prognosis of ICH.

Therefore, the purpose of this study is to investigate the relationship between poor outcome and hematoma, PHE, or total lesion volume at different time points after onset in patients with ICH. In addition, we further compared the predictive abilities of hematoma volume, PHE volume and total lesion volume for poor outcome.

## 2 Methods

### 2.1 Study Population

Between January 2015 and March 2023, we retrospectively recruited patients who were admitted to the Department of Neurology in the First Affiliated Hospital of Nanchang University. All patients received standardized clinical management according to the guideline. The inclusion criteria are: (1) age ≥ 18 years, (2) admitted to our hospital within 24 h after symptom onset, (3) completed the baseline brain computerized tomography (CT) within 24 h of onset, and (4) confirmation of supratentorial ICH consistent with the clinical deficits. The exclusion criteria are: (1) confirmation of infratentorial ICH; (2) suspected other causes of secondary ICH (coagulation disorder, trauma, tumor, central nervous system infection, aneurysms, moyamoya disease, arteriovenous malformations, etc.); (3) primary intraventricular hemorrhage; (4) subsequent surgery; and (5) lacked complete imaging, laboratory, or follow‐up records. All procedures were approved by the Ethics Committee of First Affiliated Hospital of Nanchang University.

### 2.2 Data Collection

We recorded the demographic characteristics and clinical information, including age, sex, history of hypertension, diabetes, smoking, alcohol intake, admission systolic blood pressure (SBP) and diastolic blood pressure (DBP), admission Glasgow Coma Scale (GCS), and clinical outcome (the proportion of mortality and poor outcome). A modified Rankin scale (mRS) score of ≥ 3 at 90 days after onset was defined as poor outcome (Loan et al. 2021). White blood cell (WBC), red blood cell (RBC), hemoglobin (HGB), platelet (PLT), total protein, albumin, blood urea nitrogen (BUN), creatinine, uric acid (UA), and fasting blood glucose (FBG), homocysteine, international normalized ratio (INR), and fibrinogen were measured within 24 h after admission.

### 2.3 Imaging Procedures and Evaluation

Patients underwent a standardized imaging evaluation with baseline CT scan and the follow‐up scans taken closest to 72 h (range 48–96 h) or 5–9 days after onset. We measured hematoma and PHE volumes according to a previously semiautomatical measurement (Urday et al. 2015). Briefly, the boundaries of the hematoma and PHE were outlined and adjusted in each axial slices using the Syngo.via VB20 by two neuroradiologists who were blinded to patients’ information. PHE was identified as areas of the most hypodense adjacent to the hematoma. Additionally, the region of PHE should be more hypodense than the same region contralaterally. Relative PHE was calculated by using the formula: (absolute PHE volume)/(absolute hematoma volume) (Rodriguez‐Luna et al. 2016). The total lesion volume was the sum of the hematoma volume and the PHE volume. Hematoma expansion was defined as an absolute increase > 6 mL or a greater than 33% increase between 72‐h hematoma volume and admission hematoma volume.

### 2.4 Statistical Analysis

SPSS (version 26.0) were used for all statistical analysis. Continuous variables were compared using Student's *t* test (normal distribution) or Mann–Whitney U test (non‐normal distribution) and described by mean ± standard deviation (SD) or median (interquartile range [IQR]). Categorical variables were presented as frequency (percentages) and compared using the Chi‐square test or Fisher exact test. Logistic regression models were used to identify the correlations between poor outcome and hematoma volume, PHE volume, total lesion volume, and relative PHE. Additionally, the receiver operating characteristic (ROC) analysis was performed to compare the predictive abilities of risk factors for poor outcome.

## 3 Results

**FIGURE 1:**
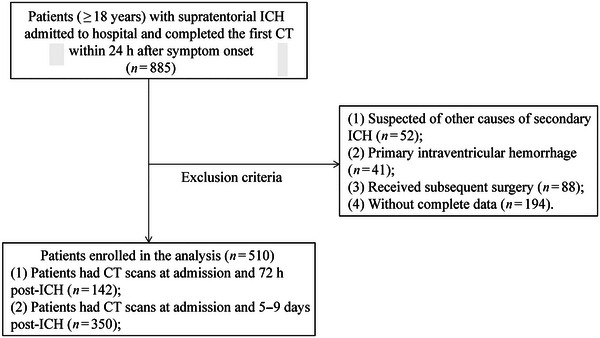
Selection of study participants. CT: computerized tomography; ICH: intracerebral hemorrhage.

Of 510 patients enrolled with incident ICH (Figure 1), 142 patients had CT scans at admission and 72 h after onset, and 350 patients had CT scans at admission and 5–9 days post ICH.

Tables 1 and  2 show the characteristics of all cohorts, patients with admissive and 72‐h CT scans, and patients with CT scans at admission and 5–9 days after onset. The median (IQR) age of all cohort was 62 (53–72) years, admission GCS 13 (13–15), WBC 7.61 (5.91–9.72) 10*9/L, RBC 4.42 (4.05–4.91) 10*9/L, HGB 133.17 (122.00–145.00) g/L, BUN 4.90 (3.70–6.00) mmol/L, FBG 5.80 (5.04–6.79) mmol/L, homocysteine 17.00 (13.00–22.06) µmol/L, fibrinogen 2.87 (2.36–3.21) g/L, time from onset to admission scan 4.07 (2.37–7.21) h, admission absolute hematoma volume 8.52 (4.31–14.57) mL, admission absolute PHE volume 9.80 (5.55–16.44) mL, admission absolute total lesion volume 18.82 (10.56–31.08) mL, and admission relative PHE 1.23 (0.82–1.74). The mean (±SD) PLT of total patients 195.84 (± 60.62) 10*9/L, and 342 (67.1%) were male. In addition, 42 (8.2%) were dead, and 213 (41.8%) experienced poor outcome among all enrolled patients. What is more, a total of 158 (31.0%) patients presented with lobar hemorrhage and 352 (69.0%) with deep hemorrhage.

**TABLE 1:** Baseline demographic, clinical, laboratory, and outcome data of patients with admission ICH scans, admission and 72‐h post‐ICH scans, admission and 5‐9 days post‐ICH scans.

Characteristics	Admission ICH scans (*n* = 510)	Admission and 72‐h post‐ICH scans (*n* = 142)	Admission and 5‐9 days post‐ICH scans (*n* = 350)
**Demographic characteristics**			
Age (y), median (IQR)	62.00 (53.00, 72.00)	64.00 (54.00, 74.00)	61.50 (52.00, 71.00)
Male, *n* (%)	342, (67.1%)	90, (63.4%)	243, (69.4%)
**Clinical data**			
Hypertension, *n* (%)	433, (84.9%)	115, (81.0%)	304, (86.9%)
Diabetes, *n* (%)	72, (14.1%)	17, (12.0%)	49, (14.0%)
Smoking, *n* (%)	108, (21.2%)	28, (19.7%)	80, (22.9%)
Alcohol intake, *n* (%)	90, (17.6%)	26, (18.3%)	66, (18.9%)
Admission SBP (mmHg), median (IQR)	150.50 (137.00, 166.00)	148.50 (137.00, 165.25)	151.00 (138.00, 165.00)
Admission DBP (mmHg), mean±SD	89.81 ± 17.09	89.27 ± 19.75	89.78 ± 16.86
Admission GCS, median (IQR)	15.00 (13.00, 15.00)	15.00 (13.00, 15.00)	15.00 (14.00, 15.00)
Admission GCS ≤ 8, *n* (%)	49, (9.6%)	17, (12.0%)	30, (8.6%)
**Laboratory data**			
WBC (10*9/L), median (IQR)	7.61 (5.91, 9.72)	7.99 (6.46, 10.35)	7.48 (5.82, 9.58)
RBC (10*9/L), median (IQR)	4.42 (4.05, 4.91)	4.38 (3.92, 4.83)	4.45 (4.06, 4.93)
HGB (g/L), median (IQR)	133.17 (122.00, 145.00)	132.00 (121.00, 141.25)	133.17 (123.00, 146.25)
PLT (10*9/L), mean±SD	195.84 ± 60.62	184.51 ± 57.54	200.41 ± 59.13
Total protein (g/L), median (IQR)	67.55 (63.40, 70.83)	67.85 (63.88, 70.70)	67.55 (63.75, 71.00)
Albumin (g/L), median (IQR)	41.00 (38.50, 43.20)	40.95 (38.30, 43.65)	41.05 (38.80, 43.05)
BUN (mmol/L), median (IQR)	4.90 (3.70, 6.00)	4.95 (3.70, 6.10)	4.80 (3.60, 5.90)
Creatinine (µmol/L), median (IQR)	69.95 (59.03, 87.08)	73.30 (61.55, 95.93)	68.20 (58.35, 85.30)
Uric acid (mmol/L), median (IQR)	321.00 (251.58, 397.20)	324.50 (246.98, 403.38)	319.00 (257.00, 393.70)
FBG (mmol/L), median (IQR)	5.80 (5.04, 6.79)	5.83 (5.12, 6.87)	5.80 (5.02, 6.86)
Homocysteine (µmol/L), median (IQR)	17.00 (13.00, 22.06)	18.00 (13.00, 22.06)	17.00 (13.00, 22.06)
INR, median (IQR)	1.00 (0.95, 1.00)	1.00 (0.94, 1.00)	1.00 (0.94, 1.00)
Fibrinogen (g/L), median (IQR)	2.87 (2.36, 3.21)	2.87 (2.39, 3.11)	2.87 (2.30, 3.30)
**Clinical outcome**			
90‐day mortality	42, (8.2%)	15, (10.6%)	19, (5.4%)
Poor outcome	213, (41.8%)	65, (45.8%)	139, (39.7%)

Abbreviations: BUN: blood urea nitrogen; DBP: diastolic blood pressure; FBG: fasting blood glucose; GCS: Glasgow Coma Scale; HGB: hemoglobin; ICH: intracerebral hemorrhage; INR: international normalized ratio; IQR: interquartile range; PLT: blood platelet; RBC: red blood cell; SBP: systolic blood pressure; SD: standard deviation; WBC: white blood cell.

**TABLE 2:** Baseline imaging data of patients with admission ICH scan, admission and 72‐h post‐ICH scans, admission and 5–9 days post‐ICH scans.

Characteristics	Admission ICH scans (*n* = 510)	Admission and 72‐h post‐ICH scans (*n* = 142)	Admission and 5–9 days post‐ICH scans (*n* = 350)
**Location of hematoma, *n* (%)**			
Lobar	158, (31.0%)	46, (32.4%)	100, (28.6%)
Deep	352, (69.0%)	96, (67.6%)	250, (71.4%)
Hematoma expansion, *n* (%)	NA	20, (14.1%)	NA
Changes of PHE from admission to follow up, mL, median (IQR)	NA	6.66 (3.44, 14.26)	8.08 (3.04, 16.25)
Onset to admission scan, hours, median (IQR)	4.07 (2.37, 7.21)	NA	NA
Onset to follow‐up scan, hours, median (IQR)	NA	65.00 (53.63, 79.56)	174.34 (150.11, 201.42)
Admission absolute hematoma, mL, median (IQR)	8.52 (4.31, 14.57)	NA	NA
Admission absolute PHE, mL, median (IQR)	9.80 (5.55, 16.44)	NA	NA
Admission absolute total lesion, mL, median (IQR)	18.82 (10.56, 31.08)	NA	NA
Admission relative PHE, mL, median (IQR)	1.23 (0.82, 1.74)	NA	NA
Follow‐up absolute hematoma, mL, median (IQR)	NA	9.17 (5.29, 17.21)	5.99 (3.03, 11.30)
Follow‐up absolute PHE, mL, median (IQR)	NA	21.64 (12.35, 33.70)	18.76 (10.83, 31.08)
Follow‐up absolute total lesion, mL, median (IQR)	NA	30.49 (17.82, 53.57)	25.6 (14.68, 43.79)
Follow‐up relative PHE, mL, median (IQR)	NA	2.15 (1.52, 3.17)	2.87 (1.93, 5.24)

Abbreviations: ICH: intracerebral hemorrhage; IQR: interquartile range; NA: not applicable; PHE: perihematomal edema.

Among the patients with admissive and 72‐h CT scans, the median (IQR) age was 64 (54–74) years, 72‐h absolute hematoma volume 9.17 (5.29–17.21) mL, 72‐h absolute PHE volume 21.64 (12.35–33.70) mL, 72‐h absolute total lesion volume 30.49 (17.82–53.57) mL, and 72‐h relative PHE 2.15 (1.52–3.17). Similarly, the median (IQR) age of patients with CT scans at admission and 5–9 days after onset was 61.5 (52–71) years, follow‐up absolute hematoma volume 5.99 (3.03–11.30) mL, follow‐up absolute PHE volume 18.76 (10.83–31.08) mL, follow‐up absolute total lesion volume 25.6 (14.68–43.79) mL, and follow‐up relative PHE 2.87 (1.93–5.24).

As shown in Table 3, multivariate regression analysis suggested that admission absolute hematoma (OR: 1.046, 95%CI: 1.010–1.083, *p* = 0.011), admission absolute PHE (OR: 1.030, 95%CI: 1.003–1.058, *p* = 0.03), admission absolute total lesion (OR: 1.037, 95%CI: 1.023–1.050, *p* < 0.001), 72‐h absolute hematoma (OR: 1.093, 95%CI: 1.002–1.192, *p* = 0.046), 72‐h absolute total lesion (OR: 1.037, 95%CI: 1.012–1.063, *p* = 0.004), 5–9 days absolute hematoma (OR: 1.061, 95%CI: 1.014–1.111, *p* = 0.01), 5–9 days absolute PHE (OR: 1.050, 95%CI: 1.027–1.073, *p <* 0.001), and 5–9 days absolute total lesion (OR: 1.053, 95%CI: 1.037–1.06, *p* < 0.001) were independently associated with poor outcome.

**TABLE 3:** Multivariate logistic regression analysis of risk factors for poor outcome.

Variable(s)	Poor outcome	
	OR (95% CI)	*p*‐value
Admission absolute hematoma^a^	1.046 (1.010–1.083)	0.011^*^
Admission absolute PHE^b^	1.030 (1.003–1.058)	0.030^*^
Admission absolute total lesion^c^	1.037 (1.023–1.050)	< 0.001^*^
72‐h absolute hematoma^d^	1.093 (1.002–1.192)	0.046^*^
72‐ h absolute PHE^e^	1.015 (0.972–1.059)	0.501
72‐h absolute total lesion^f^	1.037 (1.012–1.063)	0.004^*^
72‐h relative PHE^g^	0.802(0.578–1.111)	0.184
5–9 days absolute hematoma^h^	1.061 (1.014–1.111)	0.010^*^
5–9 days absolute PHE^i^	1.050 (1.027–1.073)	<0.001^*^
5–9 days absolute total lesion^j^	1.053 (1.037–1.069)	<0.001^*^
5–9 days relative PHE^k^	0.945 (0.886–1.008)	0.083

Abbreviations: BUN: blood urea nitrogen; CI: confidence interval; FBG: fasting blood glucose; GCS: Glasgow Coma Scale; HGB: hemoglobin; OR: odds ratio; PHE: perihematomal edema; PLT: blood platelet; RBC: red blood cell; WBC: white blood cell.

^a^ Adjusted for age, admission GCS, WBC, RBC, HGB, PLT, BUN, FBG, homocysteine, fibrinogen, time from onset to admission scan, location of hematoma, admission absolute PHE.

^b^ Adjusted for age, admission GCS, WBC, RBC, HGB, PLT, BUN, FBG, homocysteine, fibrinogen, time from onset to admission scan, location of hematoma, admission absolute hematoma.

^c^ Adjusted for age, admission GCS, WBC, RBC, HGB, PLT, BUN, FBG, homocysteine, fibrinogen, time from onset to admission scan, location of hematoma.

^d^ Adjusted for admission GCS, WBC, RBC, FBG, fibrinogen, 72‐h absolute PHE.

^e^ Adjusted for admission GCS, WBC, RBC, FBG, fibrinogen, 72‐h absolute hematoma.

^f^ Adjusted for admission GCS, WBC, RBC, FBG, fibrinogen.

^g^ Adjusted for admission GCS, WBC, RBC, FBG, fibrinogen.

^h^ Adjusted for age, admission GCS, WBC, RBC, total protein, FBG, fibrinogen, location of hematoma, 5–9 days absolute PHE.

^i^ Adjusted for age, admission GCS, WBC, RBC, total protein, FBG, fibrinogen, location of hematoma, 5–9 days absolute hematoma.

^j^ Adjusted for age, admission GCS, WBC, RBC, total protein, FBG, fibrinogen, location of hematoma.

^k^ Adjusted for age, admission GCS, WBC, RBC, total protein, FBG, fibrinogen, location of hematoma.

* *p* < 0.05.

**FIGURE 2:**
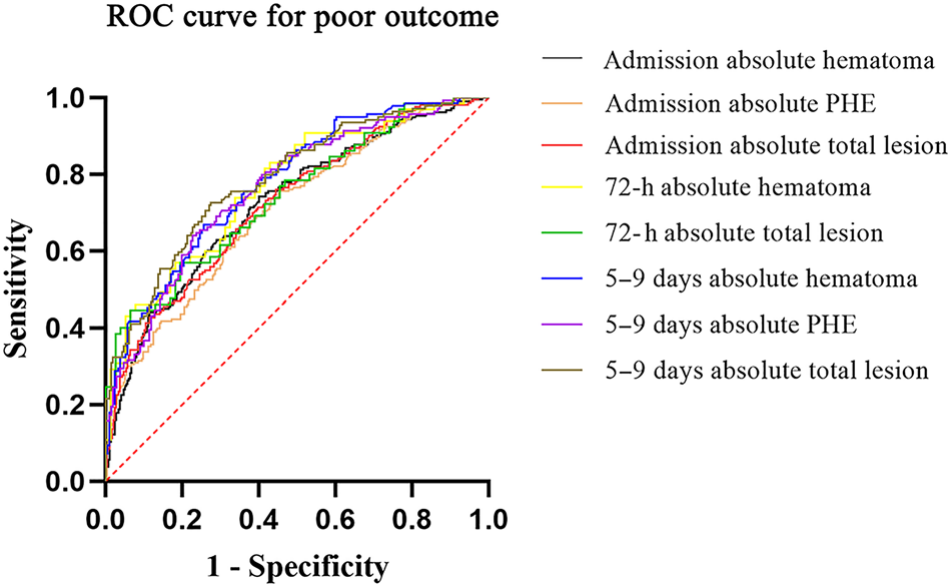
Receiver operating curve (ROC) for predicting poor outcome in intracerebral hemorrhage patients. PHE: perihematomal edema.

ROC curves were used to compare the discriminative abilities of these independent predictors for poor outcome in patients with ICH (Table 4). We observed that the area under curve (AUC) of admission absolute hematoma, admission absolute PHE, admission absolute total lesion, 72‐h absolute hematoma, 72‐h absolute total lesion, 5–9 days absolute hematoma, 5–9 days absolute PHE, and 5–9 days absolute total lesion to discriminate poor outcome were 0.725 (95%CI: 0.680–0.769), 0.706 (95%CI: 0.661–0.751), 0.724 (95%CI: 0.680–0.768), 0.769 (95%CI: 0.691–0.846), 0.742 (95%CI: 0.661–0.823), 0.778 (95%CI: 0.729–0.826), 0.765 (95%CI: 0.714–0.815), and 0.787 (95%CI: 0.739–0.836). Of note, the AUC of absolute total lesion at 5–9 days after onset was superior to other predictors in predicting poor outcome in patients with ICH (Figure 2).

**TABLE 4:** Comparison of predictive abilities of risk factors for poor outcome.

Variable(s)	*p*‐value	AUC	95%CI
Admission absolute hematoma	<0.001	0.725	0.680–0.769
Admission absolute PHE	<0.001	0.706	0.661–0.751
Admission absolute total lesion	<0.001	0.724	0.680–0.768
72‐h absolute hematoma	<0.001	0.769	0.691–0.846
72‐h absolute total lesion	<0.001	0.742	0.661–0.823
5–9 days absolute hematoma	<0.001	0.778	0.729–0.826
5–9 days absolute PHE	<0.001	0.765	0.714–0.815
5–9 days absolute total lesion	<0.001	0.787	0.739–0.836

Abbreviations: AUC: area under curve; CI: confidence interval; ICH: intracerebral hemorrhage; PHE: perihematomal edema.

## 4 Discussion

The main innovations of this study are as follows: (1) comprehensively analyze the relationship between poor outcome and hematoma, PHE, or total lesion volume in patients with ICH; (2) compare the predicted abilities of hematoma, PHE, and total lesion volume at different time points. Our results revealed that patients with poor outcome versus without had larger volumes of hematoma, PHE, and total lesion. It is worth noting that the absolute total lesion volume at 5–9 days after onset is shown to predict poor outcome in a better performance than hematoma, PHE, or total lesion volume at other time points.

It is essential to explore the predictors of poor outcome in ICH patients due to its high mortality and disability rate (Tu et al. 2023; Tsao et al. 2022). Although some traditional risk factors such as advanced age, higher NIHSS score, lower GCS score, higher blood pressure, and deep hematoma location were shown to be independent predictors of poor prognosis (Davis et al. 2006), baseline hematoma volume was considered the strongest predictor of functional outcome in patients with ICH (Broderick et al. 1993; Dowlatshahi et al. 2011). Meanwhile, hematoma expansion, which occurs in 30% of ICH patients, has also been demonstrated to be strongly correlated with dependency (Brouwers et al. 2014). In recent years, numerous studies have focused on the PHE after ICH attributing to its role in prognosis. Several studies have revealed that a higher absolute PHE volume and a larger absolute PHE growth are associated with death and poor outcomes of ICH (Yang et al. 2015; Appelboom et al. 2013; Murthy et al. 2016). Furthermore, another study confirmed the correlation between PHE expansion and mortality after adjusting for the baseline hematoma volume (Urday et al. 2016). However, most of the previous studies focused on the role of baseline hematoma or PHE volume, 24‐ or 72‐h PHE growth, and relative PHE on outcome in ICH patients. Few studies have explored the associations between the prognosis of ICH and PHE or hematoma at 5–9 days after onset. In our present study, we found that the absolute hematoma and PHE volume were the independent predictors of poor outcome, which were generally consistent with evidence from previous studies (Broderick et al. 1993; Dowlatshahi et al. 2011; Yang et al. 2015; Appelboom et al. 2013; Urday et al. 2016). However, the relative PHE at baseline, 72 h and 5–9 days after onset were all not independently correlated with poor outcome in this study. Two previous studies have found an association between relative PHE and a better functional status (Gupta et al. 2014; Gebel et al. 2002). Selection bias may contribute to our differing findings.

A previous study not only investigated the impact of PHE and hematoma on functional outcomes but also analyzed the effect of total volume on 1‐year death and dependence in ICH patients (Loan et al. 2021). Moreover, most previous studies showed that PHE and hematoma independently predicted outcomes in ICH patients. However, few studies have compared the predictive power of hematoma, PHE, and total lesion for the outcome of ICH, especially the total lesion volume at different time points. Therefore, in our study, we compared the predictive ability of hematoma volume, PHE volume, and total volume at different time points, and finally revealed the total volume of hematoma and PHE at 5–9 days after onset had the strongest predictive ability for poor outcome.

Compared with other time points, the total lesion volume at 5–9 days after ICH was the best time to predict poor outcome, and we speculate that the following reason may explain this. The development of PHE can be divided into early phase (1–4 h after ICH), intermediate phase (4–72 h after ICH) and late phase (> 72 h after ICH; Ironside et al. 2019). The PHE volume typically peaks at around 1 to 2 weeks after ICH (Volbers et al. 2016; Staykov et al. 2011). Additionally, hematoma expansion occurs mostly during the first 24 h after onset (Delcourt et al. 2017). Therefore, the maximum edema volume and hematoma volume may be present at this time point 5–9 days after ICH.

This study has some unavoidable limitations. This is a single‐center retrospective study and not every patient has CT scans at all time points, causing a little bit small sample size. In addition, although all patients recruited in our study received standardized clinical management according to the guideline, we did not include some variables such as treatment variations, rehabilitation interventions, or socioeconomic status due to the limitations of retrospective studies. Future studies should expand the sample size and complete the CT scans at all time points to confirm the results and perform the subgroup analysis for assessing the robustness of the findings.

## 5 Conclusion

In conclusion, our study suggests the total volume of hematoma and PHE at 5–9 days after onset is the strongest predictor of poor outcome in patients with ICH.

## Author Contributions

**Qin Huang**: Conceptualization; data curation; formal analysis; writing—original draft. **Lin Wu**: Conceptualization; data curation; formal analysis; writing—original draft. **Ziwei Song**: Conceptualization; data curation; writing—original draft. **Zhi Zhang**: Data curation. **Hongla Kuang**: Data curation. **Yuping Zhu**: Data curation. **Chenying Zeng**: Data curation. **Lanjiao Zhang**: Data curation. **Hudie Zhang**: Data curation. **Zubing Xu**: Data curation; funding acquisition. **Wenyuan Xu**: Conceptualization; writing—review and editing. **Jing Lin**: Conceptualization; formal analysis; writing—review and editing; funding acquisition.

## Conflicts of Interest

The authors declare no conflicts of interest.

### Peer Review

The peer review history for this article is available at https://publons.com/publon/10.1002/brb3.70340.

## Ethics Statement

This study was approved by the Ethics Committee of the First Affiliated Hospital of Nanchang University (IIT 2023 Clinical Ethic Review No. 365).

## Data Availability

Data are available upon reasonable request. All data are available from the corresponding author.
